# Pixel to Pathology: A Prospective Cross-Sectional Study on the Role of Multidetector Computed Tomography in the Evaluation of Malignant Large Bowel Lesions With Histopathological Correlation

**DOI:** 10.7759/cureus.71200

**Published:** 2024-10-10

**Authors:** Lonika Prathapan, Krishna Kumar Rama Krishnan, Priyadharshini Bala, Prabakaran T

**Affiliations:** 1 Radiodiagnosis, Mahatma Gandhi Medical College and Research Institute, Pondicherry, IND; 2 Radiology, Mahatma Gandhi Medical College and Research Institute, Pondicherry, IND

**Keywords:** histopathological correlation, lymphadenopathy, mdct, pericolic fat stranding, wall thickening

## Abstract

Background and objectives: Colorectal lesions can present as a mass or as focal or diffuse thickening of the colon wall and may also be associated with abnormalities in the perienteric region. Multidetector computed tomography (MDCT) enables simultaneous imaging of both extracolonic structures and the gut wall. It is instrumental in assessing tumor extent, detecting pericolic dissemination, including lymph node involvement, and identifying metastases. This study aims to evaluate the role of MDCT in diagnosing large bowel lesions in patients referred from the surgery department with suspected large bowel pathology and to correlate MDCT findings with histopathological results.

Materials and methods: In the Department of Radiodiagnosis at Mahatma Gandhi Medical College and Research Institute (MGMCRI), Pondicherry, this study was carried out from February 2023 to January 2024 over a one-year span. CT imaging was carried out utilizing a GE Optima 128 Slice MDCT scanner for instances of clinically suspected large bowel pathology. IV contrast, rectal, oral (positive), and plain CT were the imaging protocols that were used.

Results: Thirty of the 45 patients were female, and 15 were male; most of the patients were in the 46-60 age range. Twenty-three of the 24 instances with a CT diagnosis of malignancy had a histological confirmation. On CT, all 21 benign lesions were correctly detected. Benign lesions showed homogenous attenuation, mild symmetric wall thickening, and diffuse bowel involvement. Heterogeneous attenuation, pronounced asymmetric wall thickening, and localized bowel involvement were observed in malignant lesions.

Conclusion: For the diagnosis and differentiation of benign from malignant colon and rectum lesions, MDCT has shown itself to be an excellent technique. MDCT provides additional information about related pericolic abnormalities, lymph node presence, neighboring organ infiltration, and distant metastases in addition to identifying the lesions.

## Introduction

The second most common cause of cancer-related mortality in developed countries is colorectal cancer, a serious health concern. Age-standardized (world) incidence rates per 100,000 of colorectal carcinoma in both sexes is 19.7, in males is 23.6, and in females is 16.3 [1]. In the past, methods like an air-barium enema exam have been used for diagnosis. Colonoscopy is the reference standard for the detection of colorectal cancer and plays a major role in the diagnosis of colon cancer being the primary surveillance tool. However, the increasing use of computed tomography (CT) as the main imaging modality for gastrointestinal complaints has changed the diagnostic landscape, with radiologists frequently being the first to recommend colon cancer based on CT results.

In the world, colorectal cancer ranks second in women and third in men. Although Indian cancer registries have some of the lowest age-adjusted incidence rates of colorectal cancer globally, population-based trend studies show that the incidence of the disease is rising in India [2].

CT has proven valuable in evaluating intestinal diseases because it can assess both extra-intestinal and bowel-related diseases. It enhances diagnostic specificity by providing superior evaluations of perienteric abnormalities, including adenopathy, ascites, fat stranding, abscesses, and fistulas [3]. While barium examinations are better at evaluating intraluminal and mucosal diseases, CT excels in assessing intramural and extraintestinal components, such as the mesentery and retroperitoneum, which are vital for treatment planning. CT can be used to help diagnose other gastrointestinal disorders and differentiate them from abdominal diseases not involving the gastrointestinal system [4]. CT also allows the detection of other complications within the diverticula such as diverticular abscess, colovesical fistula, and perforation and is more sensitive than contrast enema examination [5,6].

Horton et al. in their study have said that the sensitivity of CT in the detection of primary colon cancer is variable and ranges from 48% to 77%. With better radiological staging, curative surgical resection is becoming more popular [7]. Preoperative CT provides baseline findings for comparison during the postoperative period and is the modality of choice for the detection of local recurrence after surgical resection [8].

The most common CT finding in colorectal lesions is bowel wall thickening, which presents a diagnostic challenge with its broad differential diagnosis [9]. Essential factors to consider include whether the thickening is localized, multifocal, segmental, or diffuse, along with the symmetry and enhancement patterns. Associated signs, such as lymphadenopathy or distal metastases, are also important for narrowing potential diagnoses. Contrast-enhanced CT colonography is one of the promising methods for the detection of distant metastasis. This imaging technique allowed for a more detailed evaluation of the spread of disease beyond the primary site. [10].

The feasibility of CT colonography was evaluated with a modified procedure protocol for diagnosis and cancer staging in patients with suspected acute or subacute colon obstruction caused by colorectal carcinoma [11].

Although CT is a crucial diagnostic tool, barium examinations remain important as complementary investigations in unclear cases. In addition, CT colonography has emerged as a sensitive procedure for detecting colorectal cancer, especially when combined with proper bowel preparation [12].

The prognosis for colorectal cancer is highly dependent on the stage at diagnosis, which highlights the significance of accurate preoperative staging in guiding treatment choices. Wall thickening in individuals with colorectal lesions is being classified as benign or malignant in this study in an effort to improve diagnosis accuracy by establishing a correlation between CT findings and histological findings.

## Materials and methods

Study Setting

This is a hospital-based cross-sectional study that was conducted from February 2023 to January 2024 in Mahatma Gandhi Medical College and Research Institute (MGMCRI). This study was conducted in the Department of Radiodiagnosis at MGMCRI, Pondicherry, after obtaining approval from the Ethical Committee (MGMCRI/Res/01/2021/110/IHEC/147). A 128-slice GE Optima CT scanner was used for this study.

Study design

This is a cross-sectional type of study using universal sampling.

Patient selection

Data were collected from a minimum of 45 cases of suspected large bowel lesions referred for CT of the abdomen.

Inclusion and Exclusion Criteria

The study includes patients aged 0 to 80 years who present with clinical symptoms such as abdominal distension, altered bowel habits, abdominal pain, bleeding per rectum, or weight loss. These patients must also show evidence of bowel wall thickening on a CT scan and have available histopathological findings for correlation.

However, certain groups were excluded from the study. This includes patients who have already undergone surgery or radiotherapy for bowel lesions, those for whom contrast material is contraindicated (e.g., due to renal failure, hepatic failure, or pregnancy), and patients for whom histopathological correlation is not available.

The study focuses on performing a multidetector computed tomography (MDCT) scan for patients with large bowel lesions, followed by confirmation through histopathological examination.

Data analysis

IBM SPSS Statistics (IBM Corp., Armonk, NY) was used for the data analysis. Categorical data were presented as frequencies and percentages, whereas continuous variables were described using mean and standard deviation. Calculations for diagnostic accuracy, sensitivity, specificity, positive predictive value (PPV), negative predictive value (NPV), and kappa statistics for MDCT were performed. Effect modifiers were accounted for by stratifying study variables, and the chi-square test was applied, with a significance threshold of p < 0.05.

Ethical consideration

A separate consent form was developed for each patient participating in this study. The form will outline the appropriateness and completeness of the information provided and will be approved by the local ethics committee. It will clearly explain the minor, yet above minimal, radiation risks, emphasizing that the benefits of the study outweigh these risks. Patients will be thoroughly informed about these aspects, and only after they provide their consent will they be included in this cross-sectional study.

## Results

The colon and rectum were included in the study's 45 participants who had CT images showing thickening of the gut wall. There were 15 (33.3%) ladies and 30 (66.7%) males among them. Most of the patients with colorectal lesions in our study were in the age group of 46-60 years (35.6%), followed by those in the age group of 31-45 years (28.9%) and above 60 years (20%). Patients below 30 years were the least affected, as illustrated in Table 1.

**Table 1:** Descriptive analysis of age and sex distribution (n = 45)

Category	31-45 (age)	46-60 (age)	>60 (age)	Total (age)	Female (gender)	Male (gender)	Total (gender)
Frequency	13	16	9	45	15	30	45
Percent	28.9%	35.6%	20.0%	100.0%	33.3%	66.7%	100.0%

**Figure 1:**
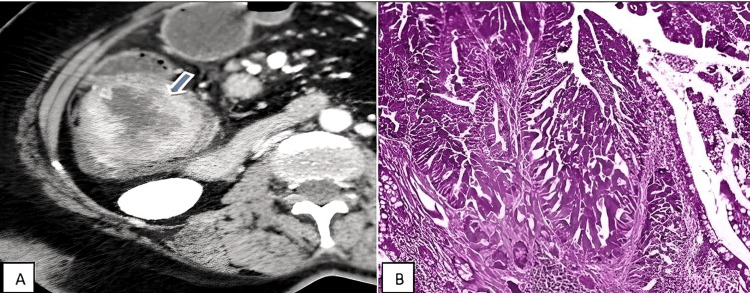
(A) Axial CECT showing heterogeneously enhancing asymmetric wall thickening (arrow) noted in the ascending colon. (B) Histopathological slide from ascending colon showing moderately differentiated adenocarcinoma. CECT: contrast-enhanced computed tomography

There was involvement of the entire length of the caecum, ascending colon, transverse colon, descending colon, and sigmoid colon in benign cases. Rectum was the most common region to be involved (47.8%) in the age group above 50 years followed by ascending colon and sigmoid colon as represented in Table 2. The involvement of the ascending colon is illustrated in Figure 1A-1B.

**Table 2:** Sites of distribution in benign (inflammatory/infection) and malignant (adenocarcinoma) (n = 45)

Location	Inflammatory/infection (n = 21)	Adenocarcinoma (n = 24)
Caecum	13 (59.1%)	4 (17.4%)
Ascending colon	14 (63.6%)	10 (43.5%)
Transverse colon	14 (63.6%)	4 (17.4%)
Descending colon	9 (40.9%)	4 (17.4%)
Sigmoid colon	4 (18.2%)	6 (26.1%)
Rectum	2 (9.1%)	11 (47.8%)

Of the 22 benign cases, 21 cases (95.45%) had homogenous attenuation and one case (4.55%) had heterogenous mixed attenuation. Of the 23 malignant cases, 20 cases (86.96 %) had heterogenous mixed attenuation and three cases (13.04%) had homogenous attenuation on CT, represented in Table 3.

**Table 3:** Descriptive analysis of the pattern of attenuation of benign (inflammatory/infection) and malignant (adenocarcinoma) lesions (n = 45) *p*-value: chi-square test

Attenuation	Histopathology		Total	p-value
	Inflammatory/infection	Adenocarcinoma		
Heterogenous mixed	1 (4.55%)	20 (86.96%)	21 (46.67%)	0.001
Homogenous	21 (95.45%)	3 (13.04%)	24 (53.33%)	
Total	22 (100%)	23 (100%)	45 (100%)	

Of the 24 cases with asymmetric wall thickening, 20 (81.8%) were malignant and four (18.18%) were benign, and of 21 cases of symmetric wall thickening, 18 (86.9%) were benign and three (13.04%) were malignant as represented in Table 4.

**Table 4:** Comparative analysis of asymmetric versus symmetric wall thickening of benign (inflammatory/infection) and malignant (adenocarcinoma) (n = 45) p-value: chi-square test

Symmetry versus asymmetric wall thickening	Histopathology		Total	P Value
	Benign	Malignancy		
Asymmetry	4 (18.18%)	20 (86.96%)	24 (53.33%)	0.001
Symmetry	18 (81.82%)	3 (13.04%)	21 (46.67%)	
Total	22 (100%)	23 (100%)	45 (100%)	

**Figure 2:**
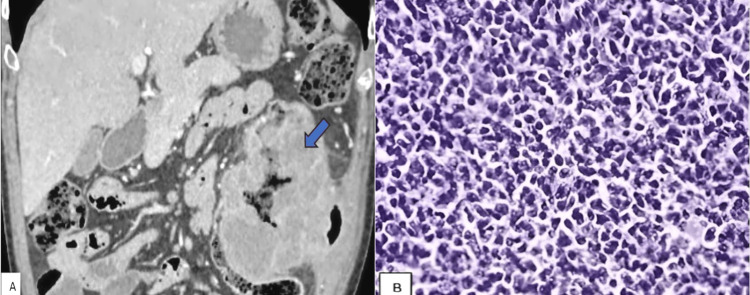
(A) Coronal reformatted CECT image showing diffuse wall thickening (arrow) noted involving the descending colon, (B) which was later proved to be lymphoma histopathologically. CECT: contrast-enhanced computed tomography

Of the 20 cases with focal involvement of the bowel wall, 14 were malignant and six were benign. Of the 11 cases with segmental involvement of the bowel, five were malignant and six were benign. Out of the 14 cases with diffuse involvement of the bowel, 10 were benign and four were malignant, as represented in Table 5. The diffuse involvement pattern of the bowel (involving the cecum and ascending colon) is depicted in Figures 2A and 2B.

**Table 5:** Comparative analysis of the length of bowel involvement of benign (inflammatory/infection) and malignant (adenocarcinoma) lesions (n = 45) p-value: chi-square test

Length of bowel involvement of the lesions	Histopathology		Total	p-value
	Inflammatory/infection	Adenocarcinoma		
Diffuse	10 (45.45%)	4 (17.39%)	14 (31.11%)	0.005
Focal	6 (27.27%)	14 (60.87%)	20 (44.44%)	
Segmental	6 (27.27%)	5 (21.74%)	11 (24.44%)	
Total	22 (100%)	23 (100%)	45 (100%)	

**Figure 3:**
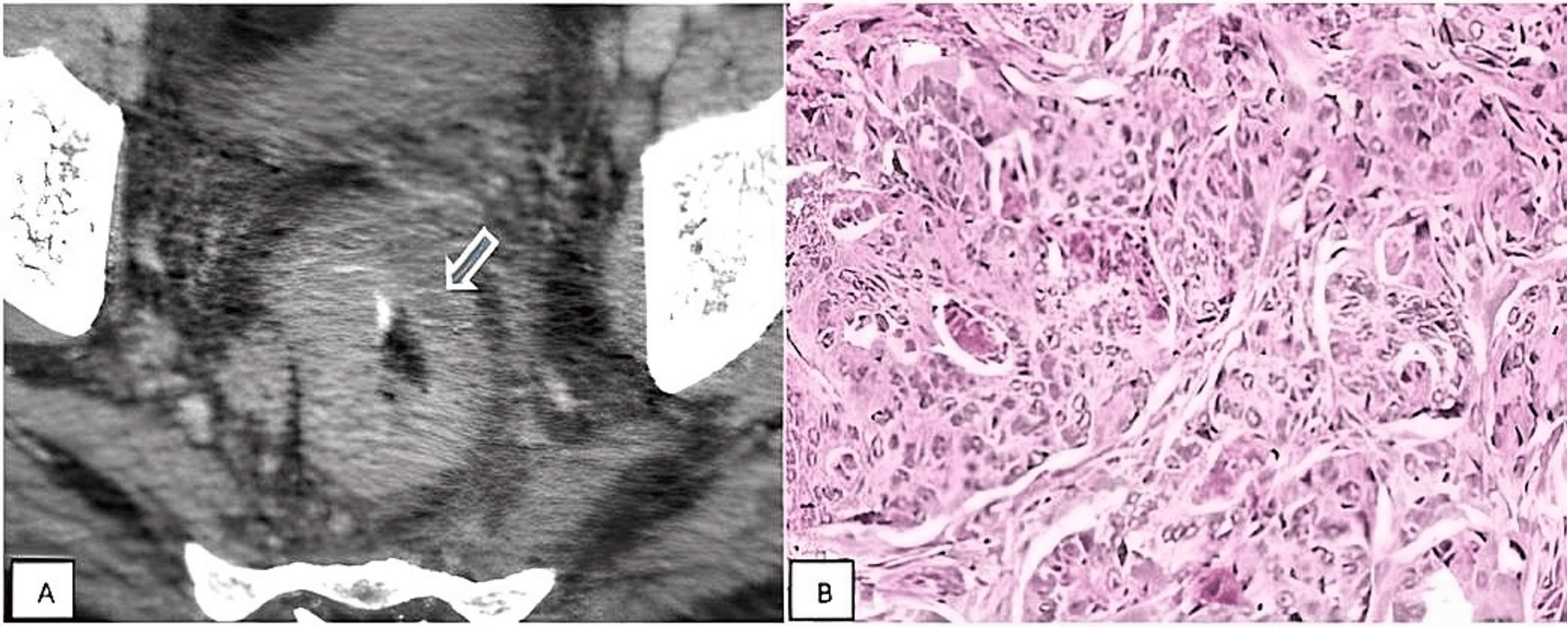
(A) Axial CECT showing heterogeneously enhancing asymmetric wall thickening (arrow) noted involving the rectum. (B) Histopathological slide showing adenocarcinoma growing in solid sheets, and a faint glandular differentiation can be noted. CECT: contrast-enhanced computed tomography

Out of 24 cases diagnosed as malignant on CT, 23 cases came as malignant on Histopathological examination and one (4.5 % ) came as benign. Out of 21 cases diagnosed as benign on CT all cases (100%) came out to be benign on histopathological examination as well, as illustrated in Table 6. Malignant lesion of the bowel (rectum) is depicted in Figure 3A-3B.

**Table 6:** Comparative analysis of CT in diagnosing benign (inflammatory/infection) and malignant (adenocarcinoma) (n = 45) CT: computed tomography, p-value: chi-square test

CT	Histopathology		Total	P-value
	Inflammatory/infection	Adenocarcinoma		
Benign	21 (95.45%)	0 (0%)	21 (46.67%)	0.001
Malignancy	1 (4.55%)	23 (100%)	24 (53.33%)	
Total	22 (100%)	23 (100%)	45 (100%)	

## Discussion

This hospital-based prospective cross-sectional study aimed to evaluate the role of MDCT in assessing malignant large bowel lesions and correlate these findings with histopathology. We examined 45 patients presenting with wall thickening of the colon and rectum, comprising 30 men and 15 women. The most affected age group was individuals over 45 years (35.6%), aligning with the study by Laishram RS et al. [13], who observed a similar pattern among 54 patients, where males (66.7%, 36 cases) were more commonly affected and most patients (24.07%) were aged 61-70 years. Of the 45 cases, 23 (51.1%) were confirmed malignant and 22 (48.9%) benign on histopathology. Abdominal pain was the most common symptom in patients with benign colon lesions, while rectal bleeding was predominant in those with malignant tumors. Benign lesions frequently involved the caecum, ascending, transverse, and descending colon, whereas malignant lesions were most often located in the rectum (47.8%, 11 cases), consistent with Laishram RS et al [13]., where rectal involvement was noted in 53.71% of the cases.

Twenty-one patients (95.45%) of the 22 benign lesions showed homogeneous attenuation, and one patient (4.5%) had heterogeneous mixed attenuation. Of 23 malignant lesions, three patients (13.0%) had homogeneous attenuation on CT, while the remaining 20 cases (86.96%) had heterogeneous mixed attenuation. This is consistent with research by Macari et al. [14] and Balthazar EJ et al. [15] that reported heterogeneous attenuation as a characteristic of malignancy (adenocarcinoma) and homogeneous attenuation as a characteristic of benign disease (inflammatory/infective).

Mild wall thickening exhibited a sensitivity of 90.9%, specificity of 82.6%, PPV of 83.3%, and NPV of 90.4% in diagnosing benign colon lesions. By contrast, marked wall thickening showed a sensitivity of 82.6%, specificity of 90.9%, PPV of 90.4%, and NPV of 83.3% for identifying malignant lesions. These findings are consistent with studies by Macari et al. [14] and Balthazar EJ et al. [15], who found that mild wall thickening is indicative of infectious and inflammatory conditions, while pronounced wall thickening suggests malignant colon tumors, such as adenocarcinoma.

The results showed that symmetric wall thickening had an 81.8% sensitivity, 86.9% specificity, 85.7% PPV, and 83.3% NPV for detecting benign lesions. This work is in concordance with Jorge Ahuhalli [16], who identified symmetric wall thickening as a characteristic of benign intestinal lesions. The results for asymmetric wall thickening were 86.9% sensitivity, 81.8% specificity, 83.3% PPV, and 85.7% NPV for detecting malignant lesions.

Of the 23 malignant lesions, four (17.3%) had diffuse involvement of the intestine, five lesions (21.7%) had segmental involvement, and 14 lesions (60.8%) had localized involvement. Out of the 22 benign instances, six cases (27.2%) had focal intestinal involvement, six lesions (26.7%) had segmental intestinal involvement, and 10 lesions (45.5%) had widespread intestinal involvement. This study is in line with the findings of Balthazar EJ et al. [15] and Macari et al. [14] who noted that localized involvement of the bowel is a characteristic of malignancy. In addition, they discovered that widespread intestinal involvement is a hallmark of an inflammatory or infectious etiology, which concurs with our study. Segmental colonic involvement, which is a characteristic of benign lesions, was present in five of the malignant cases, according to their study. The colon was segmentally involved in just 27.7% of the benign patients in our research. In light of this, our research indicates that segmental colon involvement is not a valid marker for distinguishing between benign and malignant colon lesions.

There were many enlarged lymph nodes in 88.9% of the instances involving both benign and malignant tumors. Thus, our results imply that there is no reliable way to distinguish between benign and malignant colon cancers in individuals with thickening of the colonic wall if they have enlarged lymph nodes. Hypo-attenuating bulky lymphadenopathy, on the other hand, is a supporting finding in patients with colon lymphoma, according to research by Maria et al [7]. In addition, Macari et al. [14] pointed out that tuberculosis should be suspected in low-attenuation lymph nodes with calcification or a ring of contrast enhancement.

Pericolic fat stranding was observed in all benign lesions and in 37 (82.2%) of the malignant lesions. However, eight (17.8%) of the cancer patients did not show any pericolic fat stranding. In their study, Filippone et al. [17] found that pericolic fat stranding adjacent to a malignant lesion indicates pericolic fat invasion, a characteristic of T3 lesions. However, this criterion is unreliable and may result in the overstaging of lesions as T3. In addition, Pereira et al. [18] reported that pericolic fat stranding is commonly seen in inflammatory colon conditions.

In two (4.4%) of the cancer instances, there was infiltration of the nearby structures. Cervical infiltration was observed in one patient (2.2%). In the other instance, there was infiltration of the psoas major muscle. Adjacent structural infiltration strongly suggests cancer.

In three cases (6.6%) of malignancy, distant metastases were observed. Two patients (4.4%) had liver metastases, while one case (2.2%) had lung metastases. The liver is the primary organ involved in colorectal cancer metastases [19].

Histopathology verified malignancy in 23 out of the 24 lesions (95.83%) that the CT scan had shown as malignant. Histopathology verified a CT-diagnosed malignant tumor was benign. In order to diagnose malignant lesions, CT showed a sensitivity of 100%, specificity of 95.4%, PPV of 95.8%, and NPV of 100% in our study. Histopathology verified the benign status of every instance that had been identified as benign based on CT. On CT, a lesion that was found to be malignant was found to be inflammatory during histopathological examination. Thus, for benign lesions, CT demonstrated an overall accuracy of 97.7% (showing excellent agreement) with a sensitivity of 95.4%, specificity of 100%, PPV of 100%, and NPV of 95.8%. Therefore, CT turns out to be a fantastic modality.

The limitations of this study include challenges with patient cooperation during MDCT scans, particularly in elderly or critically ill individuals, which occasionally affected image quality. In addition, some patients were lost to follow-up due to factors such as relocation or refusal to undergo a biopsy. A few participants experienced allergic reactions or contraindications to the contrast medium, resulting in incomplete or non-contrast-enhanced scans, potentially reducing diagnostic accuracy for certain lesions. The study also had a relatively limited sample size and was conducted at a single center, which may limit the generalizability of the findings to a broader population or different healthcare settings.

## Conclusions

With its ability to provide thinner sections, shorter acquisition times, and multiplanar reformatted pictures, MDCT is a highly effective modality for the identification and distinction of benign and malignant lesions of the colon and pelvis. Differentiating between early and advanced colorectal carcinoma can be done with the help of MDCT's axial and reformatted images. In addition to identifying the lesion, MDCT can reveal additional details about lymph nodes, infiltration of nearby viscera, distant metastases, and pericolic abnormalities connected to the lesion.
